# Navigating drug use, cessation, and recovery: a retrospective case notes review among sexual minority men at a community-based service in Singapore

**DOI:** 10.1186/s13011-024-00605-x

**Published:** 2024-04-16

**Authors:** Tzy Hyi Wah, Adeline Jia Xin Ong, Kuhanesan N. C. Naidu, Syaza Hanafi, Kelvin Tan, Alaric Tan, Tricia Jia Jing Ong, Eleanor Ong, Daniel Weng Siong Ho, Mythily Subramaniam, Maha Yewtuck See, Rayner Kay Jin Tan

**Affiliations:** 1 The Greenhouse Community Services Limited, Singapore, Singapore; 2 Saw Swee Hock School of Public Health, National University of Singapore and National University Health System, 12 Science Drive 2, MD1 Tahir Foundation Building #10-01, Singapore, Singapore; 3 Department of Psychological Medicine, National University Health System, Singapore, Singapore; 4 Research Division, Institute of Mental Health, Singapore, Singapore

**Keywords:** Substance use, Dependence, Holistic interventions, Recovery, Qualitative, Singapore

## Abstract

**Background:**

In Singapore, where drug use is a highly stigmatized and criminalized issue, there is limited understanding of the challenges faced by individuals, particularly sexual minority men, in their journey towards recovery from substance dependence or addiction. This qualitative study aimed to investigate the driving forces behind drug use, the factors contributing to drug cessation, and the elements influencing the recovery process.

**Methods:**

Data were extracted from clinical records provided by  *The Greenhouse Community Services Limited* between January 2020 to May 2022. These records encompassed information from four distinct forms: the intake assessment, progress notes, case closing summary, and the care plan review. Thematic analysis was employed to identify and categorize recurring themes within the data.

**Results:**

Data from beneficiaries (*n* = 125) were analyzed and yielded a series of themes related to facilitators of drug use, motivations to cease drug use, and managing one’s ongoing recovery. Within the facilitators of drug use, two sub-themes were identified: (a) addressing trauma and triggers and (b) managing emotions. Additionally, managing one’s recovery was marked by four significant sub-themes: (a) uncovering personal identities, (b) losing motivation and drive, (c) overcoming obstacles, and (d) preparing for aftercare.

**Conclusions:**

The study contributes valuable insights into the dynamics of ongoing recovery management, offering potential avenues for interventions that could enhance support for individuals in their journey to overcome substance dependence. Enhancing psychoeducation and fostering peer support have the potential to facilitate the recovery process. Clearly, a holistic approach is needed to address these complex issues that cuts across our societies.

**Supplementary Information:**

The online version contains supplementary material available at 10.1186/s13011-024-00605-x.

## Background

In public health, recovery models of substance use, and addiction are pivotal in eliciting effective treatment outcomes [28]. Recovery from substance use and addiction involves a complex process of psychological healing and transformation, where an individual overcomes substance dependence and achieves a state of improved psychological, social, and physical health [7, 54]. However, recovery models vary widely, each with its perspective on addiction recovery. For example, the *Transtheoretical Model* proposes five stages of substance use recovery that individuals experience, namely pre-contemplation, contemplation, preparation, action, and maintenance. Notably, the process of complete recovery is often assumed to be difficult and does not occur the same way with everyone [40]. Whereas the *Recovery-Oriented Systems of Care* framework focuses on systems of care that supports long-term recovery, with a continuum of services (i.e., treatment recovery support and community reintegration, [1]. Despite the diversity of perspectives on addiction recovery, scholars agree that the recovery trajectory is often non-linear, implicated by other biopsychosocial factors, and where there is no one-size-fits-all approach. Different individuals may require different strategies and support systems based on their unique circumstances and needs, that require continuous self-reflection, personal growth, and adaptation [17, 31, 53].

We note that the extant literature in addiction recovery narratives emphasizes the importance of adopting a holistic model that encapsulates individual motivations on recovery, developing healthier coping strategies, sustaining interpersonal relationships, and forging therapeutic alliances to facilitate recovery outcomes [10, 11, 47]. Despite this notion, measurements of addiction recovery outcomes often rely on recidivism data and/or drug tests [43]. These can be attributed to several reasons. First, an objective measurement, where recidivism and drug use tests provide quantifiable data that can be readily collected and analyzed. It offers concrete evidence of whether an individual has abstained from drug use, making it easier to assess progress and outcomes [30]. Second, standardization, where these assessments enable standardized measurements across different individuals and settings, reflecting the essential uniformity needed for research and policy evaluation (i.e., comparisons and generalizability of findings, [15]. Third and most importantly, legal and regulatory requirements, where court systems require drug testing as a condition of parole, probation, or participation in specific treatment regimes. Compliance with these requirements is monitored via drug test results [2]. While the reliance on recidivism data and/or drug tests provides valuable information, it is essential to recognize that these measures do not capture the entirety of the recovery process and risk oversimplifying the complexities surrounding addiction recovery. For example, underestimating drug substitution, a commonly held practice among individuals in drug use recovery, or disregarding past psychological trauma could motivate them to engage with drugs [46, 51]. To address this limitation, clinicians and researchers advocate for more comprehensive approaches to recovery measurement that combine objective data with self-reported assessments, qualitative interviews, and measures of personal growth [29, 48].

In the Singaporean context, drug use narratives are rarely explored [49]. Thus, there is a paucity of research on drug use recovery among individuals and the support systems found in community services. This may be attributed to harsh criminal penalties that create barriers for individuals to participate in related research or access healthcare resources provided by community services [13]. For example, the *Misuse of Drugs Act* criminalizes the possession and use of narcotics, including fines of up to S$20,000 to a maximum of a 10-year jail term. Additionally, the legislation mandates the death penalty or life imprisonment for trafficking in banned substances in quantities over predetermined thresholds [37]. Moreover, individuals who used or are suspected of using drugs may be subjected to investigations by law enforcement, and those who have been charged with drug use offenses could be placed under the Drug Supervision Scheme or sent to the Drug Rehabilitation Centre, which is part of the Singapore Prison Services [12].

Recreational drug use is more prevalent in sexual minority men compared to the general population [22, 41, 42]. Past studies have attributed this to issues relating to minority stress owing to one’s minority sexual orientation [16, 39]. Furthermore, research on drug use among gay men and other men who have sex with men have tended to focus on the risk factors associated with substance use, or how sexualised substance use may place them at greater risk for HIV and other sexually transmitted infections [18, 23, 50]. While some past work in Singapore has begun to detail how trauma and context serve to underpin substance use and dependence [48], to our knowledge, little to no published work has focused on the recovery journey and narratives of this population.

### Present study

To our knowledge, there is little to no published work that has formally evaluated the effectiveness of drug recovery services in Singapore. Furthermore, more research is needed on the narratives of sexual minority men undergoing recovery from drug use. This is especially salient given that the law criminalising sex between men, Section 377A of the Penal Code, was only repealed in November 2022. In light of these gaps, our study broadly explored trajectories and factors associated with drug use and the challenges individuals face in sustaining abstinence from drug use among a sample of substance use treatment-experienced clients in Singapore, who comprise largely sexual minority men. By amplifying the voices of those who have triumphed over substance use challenges, we aspire to contribute valuable insights that can inform support systems, interventions, and public health initiatives tailored to the specific needs of sexual minority men in Singapore. In this study, we extracted clinical case notes to examine the factors that motivate drug use, drug cessation, and recovery outcomes.

## Methods

### Data extraction

Retrospectively, the clinical case notes were provided by The Greenhouse Community Services Limited ("The Greenhouse) between 2020 to 2022. The Greenhouse is a community-based, charitable organization that provides peer support and substance use recovery services for sexual minorities in Singapore. Clients at The Greenhouse undergo a voluntary treatment program comprising a series of counselling sessions, engagement in a peer support programme, as well as group-based therapy and support groups. The clinical notes included from 4 different forms: *intake assessment*, *progress notes* (PN), *case closing summary* (CCS), and *care plan review* (CPR). These forms were completed in English language by licensed counsellors.

All clients who initially arrive at The Greenhouse would fill in the intake assessment form. Thereafter, they can choose to attend various in-house programs, such as group therapy, individual counselling, or peer support – whichever best suits the client’s recovery needs. The intake assessment form contained information on client’s background, chief concerns (CC), and a brief trauma description. Responses gathered from the CC section were guided by three items: *what brings you here today?*, *how can we help you?*, and *what is your treatment goal?*. CC data were used to uncover the themes associated with drug use motivation and client’s motivation toward recovery.

Further, PN and CCS were made available only to clients who decided to attend in-house counselling programs. PN, in particular, contained information about client’s recovery struggles, recovery goals, coping strategies employed to deal with drug use temptations, and personal milestones during recovery. Besides, CCS data provided a succinct summary of the client’s recovery journey and their anticipated needs for continued clinical care. Notably, CPR data is made available to clients who wish to review their recovery progress after completing or terminating their participation in the in-house programs. These included post-program information on the client’s protective and risk factors for drug use and a review of current recovery progression.

### Data analysis

A *thematic analysis* was conducted to code and analyze the clinical case notes following Braun and Clarke’s (2006) six-step framework. In the first step of familiarization with data, our researchers sought to read through all the clinical case notes from participants to get a stronger sense of the data. In the second step, the research team started initial coding of the clinical case notes without any pre-existing framework and starting developing categories of similar codes. It was at this stage when codes like ‘self-security’, ‘setting healthy boundaries’, and ‘fears and worries’ were generated. In the third step of searching for themes, we then sought to further categorise our codes and generated broader narratives that reflected and aligned with participants’ recovery journeys, which generated phrases like ‘factors influencing drug use’. Weekly meetings were held to explore different categories and themes from the data. In the fourth and fifth steps of reviewing and defining themes, we then continued to gather more data and codes and compared this to the broader framework of themes that we had generated to ensure that the themes made sense, and that the data supported the themes. While theme descriptions were developed, the team concurrently consulted extant literature to refine these themes [9]. This was when we generated two separate, higher-order themes of ‘factors influencing and stopping drug use’ (Table 2), and managing one’s recovery (Table 3). In the final step, we then reporting our findings in this manuscript [8]. Besides, a case study method was adopted to better illustrate an in-depth appreciation of an individual’s recovery progress. A case study was selected based on the completeness of the clinical data (i.e., no missing information in PN, CC, CCS, CPR) and a case that best signifies the recovery process of The Greenhouse's clients [44].

### Reflexivity and positionality

**Fig. 1:**
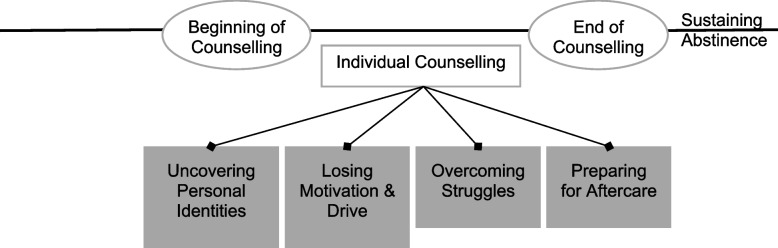
Client’s recovery process

Qualitative research studies rely significantly on the nuanced judgement of the researcher. Therefore, we acknowledged that reflexivity and the constant need to consciously critique and evaluate one’s work is critical in generating unbiased qualitative data [4]. The clinical data for this study were obtained from a team of trained counsellors (with clinical supervision) at The Greenhouse. The data were subsequently analyzed by two research assistants (RAs) who then worked with counsellors to develop the findings of this study. Both RAs were undergraduates with psychological and/or life sciences research experiences. Therefore, the research team’s diversity contributed fresh perspectives to the study. Additionally, the collaborative relationship between stakeholders allowed for better comprehension of expression or content from the clinical notes with an understanding of terminologies, sensitivities, and concerns around individuals in addiction recovery. Conversely, this bore limitations on the scope of data generated and analyzed in the study because the clinical notes obtained were based on the counsellors’ interpretation of the CCS, trauma, and concerns that may simplify the recovery narratives. Therefore, to preserve the originality and validity of the clinical notes, multiple discussions and reviews were held among a diverse team of researchers Fig. 1.

### Ethics approval

This study was approved by the National University of Singapore Institutional Review Board (Reference: NUS-IRB-2022-457). Participants provided written documented consent for the use of their data for research purposes during their intake to the community-based program.

## Results

### Clients’ demographics

Table 1 illustrates a summary of the clients’ demographic characteristics obtained from (*n* = 125) intake assessment forms. Majority of the clients identified as sexual minority men, held Singapore citizenship, and were between the ages of 30 to 39. Further, most have obtained a bachelor’s degree and are of Chinese ethnicity. Additionally, clients reported identifying with various religious affiliations, where a large proportion are *Free Thinkers*. While most clients live with their families, do not have HIV, and have never been incarcerated, many have had a substance use history and used four types of substances (polydrug use) on average. Overall, most clients sought treatment via community treatment programs, where 35.2% attended individual counselling after intake assessment was completed. Notably, most clients reported abstinence as their recovery goal.

**Table 1:** Client’s demographics from the intake assessment forms

Age (M(SD))	20 to 29: 35
	30 to 39: 55
	40 to 49: 28
	50 and above: 5
	NA: 2
	Mean: 34.7
Gender n(%)	M = 123
	F = 1
	NA = 1
Sexual Orientation n(%)	Bisexual: 10 (8.0)
	Gay: 108 (86.4)
	Pansexual: 1 (0.8)
	Straight: 4 (3.2)
	Uncertain: 2 (1.6)
Nationality n(%)	Foreigner: 9 (7.2)
	PR: 9 (7.2)
	Singaporean: 107 (85.6)
Ethnicity n(%)	Afghan: 1 (0.8)
	Chinese: 88 (70.4)
	Indian: 12 (9.6)
	Latin: 1 (0.8)
	Malay: 17 (13.6)
	Mixed: 3 (2.4)
	White: 3 (2.4)
Residence status n(%)	Alone: 25 (20.0)
	Family: 83 (66.4)
	Friends: 3 (2.4)
	Housemates: 3 (2.4)
	Partner: 8 (6.4)
	Tenants: 3 (2.4)
Educational attainment n(%)	Graduate: 59 (47.2)
	Postgraduate: 15 (12.0)
	Secondary: 8 (6.4)
	Tertiary: 41 (32.8)
	NA: 2 (1.6)
Ever incarcerated n(%)	No: 84 (67.2)
	Yes: 39 (31.2)
	NA: (1.6)
Experience with SUD treatment n(%)	No: 55 (44.0)
	Yes: 69 (55.2)
	NA: 1 (0.8)
HIV and other STI Status n(%)	No: 60 (48.0)
	Yes: 51 (40.8)
	Uncertain: 11 (8.8)
	NA: 3 (2.4)
Religion n(%)	Buddhist: 19 (15.2)
	Christian: 29 (23.2)
	Free Thinker (+Agnostic, Atheist): 47 (37.6)
	Islam: 20 (16.0)
	Others: 9 (7.2)
	NA: 1 (0.8)
Mean no. of substances used	4.16129
Clients who went for individual counselling n(%)	44/125 (35.2)
Recovery goals*	Reduction: 2 (1.6)
	Abstinence (including those who indicate long term abstinence): 60 (48.0)
	Relapse prevention: 11 (8.8)
	Unknown: 52 (41.6)

### Factors facilitating drug use

Across clients, the two themes found to significantly influence their decisions to use drugs are illustrated in Table 2, which include ‘managing trauma and trauma triggers’, and ‘managing feelings and emotions’. Generally, the reasons for drug use are individual focused, where clients are unable to cope with their past traumas, trauma triggers, and their associated emotions. For example, clinicians made a brief note on specific factors that potentially threaten a client’s sobriety: “*family related, sex, triggers, trauma*.” This was an example of clients having to manage trauma and trauma triggers, with specific indications that the client faced issues around managing their own family relationships, their relationship between sex and drugs, dealing and navigating triggers for trauma and substance use, as well as trauma underpinning their substance use.

**Table 2:** Clinical excerpts of clients’ chief concern depicting their motivation to use and stop drug use

**Category**	**Theme Title**	**Theme Description**	**Clinical Excerpts**
Factors influencing drug use	Managing trauma and trauma triggers	When clients mention that they are using drugs as a means to cope with their past trauma and their triggers.	• “Client wants to hear from experiences of fellowship on how to handle triggers, cravings etc.” • “He thinks issue comes from psychological aspect that could not articulate” • “Family related, sex, triggers, trauma”
	Managing feelings and emotions	When clients mention that they are using drugs to cope with feelings, such as anger, boredom, shame, etc.	• “Use drug to escape boredom, shame of christian gay” • “Acting out angrily when using substance” • “Deal with feelings of guilt”
Factors stopping drug use	Managing relationships	When clients mention that they are hoping to stop using drugs to improve the quality of relationships around them, such as with friends and family members.	Friends • “Relationship - can cause triggering when fighting” • “Repair rupture in the relationship.” • “Problems making friends and having close friends” Family • “Find ways to build trust with mother (mum's anxiety with his using)” • “Affected r/s with partner… …He hates the effect of using. Miss out a lot in loved ones' life... Does not want to lose family.” • “Disclosing and coming to terms with family”

Data were taken from the Chief Concerns of the Intake Assessment Form (CC), *n* = 125

### Motivations to cease using drugs

Conversely, the desire to improve the quality of current relationships tended to motivate clients to cease their drug use. As illustrated by clinical excerpts, “*problems making friends and having close friends*”, these factors led clients to abstain from drugs. Besides, friendships, relationships among family members were strong motivators for ceasing drug use. Further, a clinician noted the detrimental effects of drugs on a family as a client mentioned how using drugs have negatively impacted his relationship with his loved ones: “*Affected r/s (relationship) with [partner] … He hates the effect of using [drugs]. Miss out a lot in [loved ones’] life… Does not want to lose family.*”

### Managing one’s ongoing recovery

Table 3 illustrates some themes in the recovery process that were highlighted by clients in general and in the selected case study. Supplementary Table 1 provides descriptions of each category and theme. Drawing from the case study, the client’s recovery progress started with themselves by uncovering personal narratives. This included their experiences with the multiple identities they possess, such as being a son, a husband, and a partner. Further, losing motivation and drive for recovery surfaced when they experienced circumstances that threatened the client’s reason for sustaining abstinence. This included feelings of loneliness, because no one understands their situation; hopelessness, because they do not see how abstinence is possible; and a lack of belonging because clients were unable to feel as though they were part of a social group, because appearing as an individual recovering from drug use was perceived to be *unacceptable.*

**Table 3:** Categories and themes generated from clinical excerpts of clients’ chief concerns, case closing summaries, and progress notes which illustrate their recovery

**Category**	**Theme Title**	**Theme Description**	**Clinical Excerpts from the Case Study (PN)**	**General Clinical Excerpts Supporting the Theme**
Uncovering personal identities	Identifying and forming a cohesive personal narrative	Clients shares about their roles in their birth and/or affinal families (e.g son, husband, partner) as well as other identities they choose to describe themselves with (e.g people who use drugs, gay man).	• Juggling “multiple selves”. • Identified fear of being “soft” and “yielding”, and desire to honour father’s disciplining • Examining values in coming out to family	• Feels guilt over drug use: “Let my parents and family down” (CC)
Losing motivation and drive	Feeling lonely or isolated	When clients mention that they feel isolated or alone, and want to work through their thoughts and feelings in relation to loneliness. This may also be the case when clients still interact with others within their social circle.	• Acknowledged emotional avoidance, fear of loneliness.	• Unable to talk to friends about his addiction, no one understand his addiction situation (CC) • Lost, loneliness, low self-esteem, Family recently found out he is seeing a psychiatrist (CC) • To address fear of being alone, wanting to be around people (CC) • Feels lonely, need to develop support system (CC)
	Feeling hopeless about recovery	When clients express emotions related to a combination of negative life events, and thought patterns, particularly those of self-blame and expressing the idea that circumstances are unchangeable.	• Addressed harsh self-criticism over “bad self” • Losing motivation to change • Explored ambivalence about change	• "I want to stop using substance completely but I don't think it is even possible” (CC) • Feelings of despair…Concerns about therapeutic progress (PN) • Losing motivation to change (PN)
	Lack of belonging with others	When clients express their want to be present with others (example: friends, family, partner) without sacrificing who they are. This also implies that there is a mutual agreement between the social group’s wants to be present with the clients.	• Need to review relapse management and relationship with peer supporter. • Planned new resources in social support and stabilisation • Acknowledged value of collaboration	• Want to find a community that he can be himself. (CC) • Client celebrated his achievements in the last 9 months, and also identified needs for love & belonging currently not satisfied due to him not taking action to rebuild his relationship. (CCS) • Client mentioned that he felt more fulfilled and at ease with himself, and is in a relationship where he feels love and closeness. (CCS)
Overcoming struggles	Attempting to make peace with their past experiences (either using drugs or traumas)	When clients process through feelings and emotions from past negative experience or traumas. In doing so, these past experiences do not greatly impact current experiences	• Honoured integration of good/bad new/old parts • Examined childhood trauma, and self-image as “monster” with dark side	• Client was able to identify importance of experiential contact with emotions and was able to engage in healthy confrontation and processing of emotions in response to grief and early traumatic experiences (CCS) • Client was able to process his childhood trauma and reduce the frequency of negative thoughts associated with guilt and self-blaming. (CCS) • Reflected on how past traumas and present triggers affect decision and wanted to have longer “clean” time to be credible enough in order to serve the recovery community. (CCS)
	Self-development • Self-security • Self-awareness • Self-esteem • Self-efficacy	When clients share about some things that they hope to work on, in order to better themselves *Self-security: When the clients express the want to have an open and non-judgmental acceptance of their own weakness. *Self-awareness: When clients express that they want to better identify their state and emotions in the present moment. *Self-esteem: When clients assess people and what they have accomplished to their values and goals and express their wants to boost their confidence and self-esteem. *Self-efficacy: When clients express their want to be self-sufficient or self-sustaining to reach specific goals.	Lack of self-security • Psychoeducation on self-acceptance and motivation. Challenging good/bad new/old dichotomy • Amplified ambivalence over self-acceptance • Increased self-blame Improving self-awareness: • Developing curiosity about behaviours. • Recognised less avoidance of sorrow and more “slowing down” Building self-esteem: • Re-valued humour, honesty, freedom. • Experiencing joy in values. Self-efficacy • Self-compassion practice for self and other • Balanced self-doubt with self-kindness and humour. • Honoured integration of good/bad new/old parts	Self-security • Client’s health and wellbeing have improved over the sessions and is starting to accept herself inside and out for who they are (CCS) • He became more compassionate to his own needs for safety and connection (CCS) • Client was better able to work on responding to emotions better, and through honesty client was able to be himself, relieving himself from having the need to be someone he is not through lies. (CCS) • Client remove the facade of upkeeping image and understood the true meaning behind not good enough. (CCS) Self-awareness • Client was also aware of his risk-avoidant behaviour in building romantic relationships. (CCS) • His self-awareness has increased, particularly how his strong values may impact his relationships with others. (CCS) • Significant improvement in building awareness and managing emotions, resulting in a greater sense of calm and less rebuttal of ideas Self-esteem • His self-esteem and confidence have grown and has a greater awareness of how his thoughts influence his emotions and behaviours. He is receiving recognition for his work which has helped his self-esteem. He shared his aspirations and left the last session full of hope and confidence in his ability to achieve these. (CCS) • Build a support system with fellows at the greenhouse and worked his way to service in the peer support meetings through building rapport and gaining self-esteem. (CCS) • The client wishes to focus on providing a service to others and continues to build confidence through co- facilitating, and on occasions leading, group sessions at The Greenhouse. (CCS) Self-efficacy • Wanted to ask for help but too ashamed to ask around and do not know where also, worried that he goes to wrong place for help authorities. Wanted to find a place without judgement so he can ask for help. (CC)
	Setting healthy boundaries	When clients want to create a space where they can love themselves and others simultaneously. This includes taking another person’s feelings into consideration, setting clear limits and showing mutual respect [18].	• Exploring value in relationship boundaries. • Clarifying relationship boundaries and needs. • Examined relationship boundaries and expectations in family	• At the end of the 10 sessions, he was functioning well at work and managing to set healthy boundaries within relationships. (CCS) • Client has also grown in confidence and is setting healthy boundaries in not getting too involved in supporting others which they used to do at the detriment of their own health and wellbeing. (CCS) • He clarified some relationship boundaries with his partner, by defining his locus of control and expressing his needs more clearly. (CCS)
	Effective communication and understanding	When clients express a desire to improve their communication with another person. Clients would ideally exchange thoughts and opinions to the other person so that the message is conveyed and understood with clarity and purpose.	• Clarified ambivalence over partner’s new independence • Coming out to children	• His ability to communicate more effectively with parents had also improved. (CCS) • The client is more reflective and patient with others, in particular with his family. (CCS) • Client was taught coping strategies to build resilience and reduce stress and anxiety levels which included breathing techniques and psycho education on transactional analysis to help with communication with parents. (CCS)
	Feelings of discrimination	When clients feel discriminated for being who they are. These can be in relation to their sexual identity, religious affiliations, being HIV positive or as a known person who uses drugs. As a result, they experience feelings of shame, disgrace or dishonour.	• Acknowledged guilt from “objectively disordered” label • Pressure from others to be “cured”	• Use drug to escape boredom, shame of being Christian and gay (CC) • To come to terms with sexual orientation and HIV status (CC) • Wanted to ask for help but too ashamed to ask around and do not know where also, worried that he go to wrong place for help authorities. (CCS) • Want to be in a relationship, fearful due to HIV status (CC)
	Helpful techniques and exercises	When clinicians note down some techniques or exercises that clients work on during the course of counselling	• Psychoeducation on trauma’s impact and therapy process • Designed experiment in “showing up” when avoidant • Role-play on responses to pressures • Psychoeducation on phases of change and termination	• Better coping mechanisms: Meditation, aexercise, dancing, socializing with friends. Internal locus of validation: Daily gratitude to move away from what client doesn’t have. Cognitive defusion: unhook from negative emotions. (CCS) • Turned towards more meditation and taking heed of space and pace. (CCS)
Preparing for aftercare	Re-evaluating values and goals	When clients reflect on current or past goals in relation to their counselling aims	• Clarifying value of authenticity to self. • Re-valued “showing up” as vulnerable self for interviews and recovery meetings.	• Was able to experiment with different strategies to manage thoughts, feelings and behaviours with success. Was able to reconceptualise his understanding of his values. • He also practised and internalised ACT [acceptance and commitment therapy] skills to promote values-guided behaviour.
	Preparing for post-therapy	When clients share about their plans and preparations for post-counselling	• Co-designing termination activity. • Experiencing fear of termination	• Training to become a peer supporter (CCS) • Client is committed to his work as a peer supporter, which enables him to contribute meaningfully to others and practise living with triggers in his relapse recovery. To manage challenges in this process, he is willing to seek support from other peer leaders. (CCS) • Client can access other Greenhouse relevant services if needed (i.e., Smart Recovery; Narcotics Anonymous) (CCS)

Data extracted from the Chief Concerns of the Intake Assessment Form (CC), Progress Notes (PN), and Case Closing Summaries (CCS)

Taken together with external stressors (i.e., work and relationship tensions), the themes of losing motivation were identified as risk factors that facilitated relapse, self-doubt about one’s recovery outcomes, and discouraged clients from pursuing recovery. As the client identified obstacles and setbacks to recovery, he was able to work them through with his counsellor and incorporated the skills learnt from counselling into his everyday life. Notably, feelings of discrimination were a significant theme that stood out as an obstacle in the recovery process.

Towards the end of counselling, the counsellor and client in the case study shifted the sessions’ focus toward re-evaluating values and goals, specifically, plans that the client had for aftercare. At this instance, the clinician noted that the client expressed fear about the end of counselling. Following this, the client was empowered to co-design their termination activity. Other clients expressed their desire to contribute back to The Greenhouse by serving as peer supporters. Supplementary Table 2 further illustrates the comprehensive progress of notes attained for the case study.

## Discussion

The present study is the first to explore factors that facilitated drug use, motivated ceasing one’s drug use, and managing one’s ongoing recovery among clients who utilized community services at The Greenhouse in Singapore. Of note was that the trajectory around managing one’s recovery tended to transverse several subthemes, from uncovering personal identities, losing motivation and drive toward recovery, overcoming struggles, to preparing for aftercare (Figure 1). As noted in previous literature, clients may regress to earlier stages of recovery when they experience a relapse [4, 40]. Consistent with our case study, the client struggled to work through his personal identity, relationship strains, and other external stressors. Therefore, clients tend to be more vulnerable to potential relapse as they navigate through the first three stages of recovery.

As illustrated in Table 2, one of the prominent motivations that drove continued drug use was to manage one’s feelings and emotions. A reason why clients could be more prone to relapse was that they relied on drugs as a maladaptive coping strategy, including avoidance coping strategies aimed at reducing or avoiding immediate distressing experiences [14]. Consistent with alcohol use literature, evidence suggests that individuals who rely on avoidance coping strategies are more likely to engage in problematic drinking behaviors [52]. Generally, reasons for clients to engage in drug use vary from being bored to managing anger, guilt, and shame. For sexual minority clients, the intersectionality of their identity becomes particularly crucial in understanding how these motivations manifest. The lived experiences of being a sexual minority can contribute to heightened psychological distress due to minority stressors, potentially influencing substance use patterns [35, 36]. Stigma related to sexual minority identity, as identified in our case study, emerges not only as a motivator for seeking recovery but also as a significant threat to the recovery process. Clients grapple with internalized, enacted, or anticipated stigmatized labels, such as being a person who uses drugs or a sexual minority, adding an additional layer of complexity to their recovery journey. Hence, rendering these individuals at greater risk of substance use and dependence [48, 49].

Drawing from the case study, feelings of loneliness and hopelessness appeared as prominent themes that were identified when the client lost motivation and drive for recovery. Loneliness as a subjective experience is associated with poor physical and psychological health outcomes [45]. Further, the general excerpts and case study identified *feeling lonely* as a risk factor for drug use relapse. This aligns with the extant literature investigating the relationship between loneliness and drug use and found that loneliness is a significant risk factor for drug use [21, 26]. Nevertheless, this relationship could be moderated by the type of coping styles employed by individuals [34]. Conversely, hopelessness increases when clients face a series of adverse events and believe that recovery is impossible. When revisiting the qualitative data, the theme of hopelessness is identified in the earlier stages of recovery. Further, feelings of hopelessness were identified in our case study, where the client navigated through a breakup, worsened familial tensions, and managed feelings of inefficacy.

Moreover, for sexual minority clients, hopelessness takes on a distinctive dimension, influenced by adverse events and the overarching belief that recovery may be an insurmountable feat. It is crucial to acknowledge the role of being a sexual minority within this dynamic, as the added layers of stigma and societal pressures may intensify feelings of hopelessness. Therefore, our study accentuates the need for a more nuanced exploration of how being a sexual minority intersects with emotional states like loneliness and hopelessness, shedding light on the distinct factors that contribute to the recovery challenges faced by this marginalized population.

Notably, this study identified the significance of *social belonging*, pertinent to the recovery process, specifically in the aftercare phase. A *sense of belonging* refers to an individual’s perception of *relatedness* to a social group [33]. While true belonging demands authenticity and courage from individuals to show up as themselves, it could be extremely challenging for individuals in recovery from drug use in *drug-free* societies to achieve [9]. A potential explanation would be that belonging is enmeshed within the broader socio-political landscape that highly stigmatizes drug use [3]. For clients at The Greenhouse, commonly stigmatized labels that were internalized, enacted, or anticipated include being a person who used drugs, sexual minority, or living with HIV. Interestingly, stigma was identified as a motivator for clients to seek recovery and a threat to a client’s recovery process. For clients who expressed their desires to reconnect with others who do not engage in drug use, being able to show up as an individual in recovery could be a vulnerable and uncomfortable experience. To make matters worse (since drug use is highly stigmatized in Singapore), clients often face social rejection from loved ones who do not engage in drug use. Therefore, the social pressure to fit into the drug-free narrative and the desire to reconnect with others could have motivated clients to seek out drug recovery services [5]. With that in mind, a client’s desire for social belonging can further hinder his/her recovery process especially in the Singaporean context, where harsh criminal penalties create barriers for individuals access healthcare resources provided by community services [13]. This fear of once again having to experience rejection from their loved ones and social network could be bolstered and demoralize clients from working toward recovery. Conversely, re-engaging in substance use behaviors could reinforce a client’s sense of belonging. For example, intimacy could be achieved with sexualized drug use, or as a way of socially engaging with peers [48, 49]. Overall, clients who are caught in between social connections who both engage or do not engage in drug use will have to eventually choose between going through recovery or not, which potentially risks losing valuable relationships.

More importantly, our qualitative data supported the notion that clients required various social groups to achieve their recovery needs. As illustrated in Table 3, when clients completed the intake assessment, they expressed assistance-related concerns, such as guidance, non-judgmental support services, and discussions on navigating social relations. For example, clients who received validation for their recovery process reported feeling valued and confident in achieving abstinence. They subsequently indicated an interest in wanting to give back to society by becoming peer supporters towards the end of counselling sessions, further suggesting that social support serves to bolster self-confidence and empowers clients in addiction recovery. This is consistent with Hall et al.’s [20] and Ogilvie and Carson’s [38] studies that demonstrated clients who were perceived as *competent* in addiction recovery were less likely to relapse in the future. Similarly, the subjective perception of recovering clients could have uplifted their self-confidence, motivating them to overcome their addiction issues [6].

Moreover, a significant part of overcoming recovery struggles requires clients to internalize and work on their personal growth (i.e., self-awareness, self-esteem, and self-security) and reinforce coping skills [5]. These reframed thoughts are pertinent in helping clients increase their self-efficacy with the belief that they can sustain recovery beyond counselling [24, 55]. As illustrated in Table 3, general excerpts revealed that achieving a level of personal growth positively influenced the client’s recovery journey. Notably, these clients possessed the ability to acknowledge their strengths, weaknesses, values, and the awareness of maladaptive behaviors in times of distress. Clients acquired a more objective view of themselves as a result, which may in turn, increase their self-efficacy. This is consistent with Kang et al.’s [25] study which demonstrated that journey toward drug use recovery is a process of personal growth (and not just abstaining from drugs).

Lastly, psychoeducation appeared to be a significant element in counselling. As illustrated in Table 3, psychoeducation materials were tailored based on the clients’ needs ranging from designing and attempting exposure-based experiments, and distress tolerance coping skills, to integrating healthy and sustainable habits into clients’ everyday lives. Clients are taught about their drug use tendencies, trauma symptoms, practice various coping skills, and share their struggles in addiction recovery. Thus, psychoeducation allows clients to reflect on their experiences through a psychological lens and develop appropriate tools to aid them in their recovery journey. As mentioned above, sustaining abstinence from drug use is only one of the many struggles faced by clients in recovery. This demonstrates the importance for clients to take ownership of their recovery needs and develop/re-design a variety of healthy yet sustainable skills when needed. Encouraging clients to share their struggles in addiction recovery within the psychoeducational context becomes particularly significant for sexual minority individuals. By fostering an open and supportive environment, psychoeducation can empower clients to address the specific challenges they face due to their sexual minority identity. This collaborative approach enhances the effectiveness of psychoeducation and contributes to a more inclusive and tailored recovery process.

Limitations of the present study should be noted. First, the qualitative data consisted of clinical case notes that were summarized by counsellors. This introduces inaccuracies in data interpretation and may not necessarily reflect the emotions and feelings of clients accurately. Second, our study did not uncover the influence of individual difference mechanisms on recovery (i.e., social support and stigma). This could potentially overlook unique nuances that may impact a client’s journey in recovery. Third, clients who came forth to access clinical services at The Greenhouse could likely represent a specific population within sexual minorities in addiction recovery (i.e., largely gay men, and higher education attainment). As such, the results may not be generalizable to other sexual minorities (lesbian women, transgender individuals, etc.). Furthermore, the narratives are reflective of individuals who have taken a step forward in seeking help for their substance use, and may not be generalizable to the wider population of people who use drugs. Taken together, it is recommended that future qualitative studies explore the influence of individual difference mechanisms, including a wider sexual minority population, and possibly the perspectives of primary caregivers, and the barriers they face in supporting individuals in addiction recovery. Further, it would be of clinical interest to explore the psychotherapeutic processes at play in drug use cessation and the clinical management of recovery outcomes to improve addiction services in community organizations.

### Implications and future directions

With the results and limitations in mind, the strength of the present study is bolstered by its novelty in attempting to uncover factors that influence addiction recovery in the context of sexual minorities in Singapore. The study uncovered that willpower by itself is insufficient to maintain sobriety since there is a complex relationship between the motivations for drug use and drug cessation. Further, the findings revealed individuals who engage in drug use contend with realities, such as prejudice against sexual minorities and drug use behaviors, both of which impede their ability to recover. Therefore, our study demonstrated the importance of delivering holistic programs for clients in addiction recovery, specifically a model that emphasizes personal growth, social support structures, and healthy coping mechanisms to empower them to take ownership of their recovery journey.

Notably, there are further implications arising from this study that could be of relevance to the community, mental health practitioners, and policymakers. First, in communities, it is crucial to provide additional opportunities that prioritize judgement-free care for individuals in drug use recovery. Moreover, communities must confront the stigma they often attach to individuals who engage with drugs and understand that these negative connotations can pose significant barriers to those seeking recovery services. Rather than isolating or stigmatizing these individuals, friends and family members could strive to empathize with those in their recovery journey and provide social support toward recovery [19, 27, 32]. Second, mental health practitioners play a pivotal role in aiding individuals in drug use recovery to identify and address their struggles. Additionally, practitioners could highlight key personal growth milestones in their client’s recovery journey, which may, in turn, encourage these individuals to continue persevering through continued recovery. Importantly, this emphasizes the need for culturally competent clinicians who can provide clinical care specific to sexual minority individuals in addiction recovery. Third, from a policy perspective, recognizing the multifaceted nature of factors impacting the recovery process underscores the necessity of formulating comprehensive metrics for the identification of vulnerabilities associated with drug relapse.

In a similar vein, given the diverse range of factors influencing addiction recovery within the context of sexual minorities in Singapore, future research should explore the potential utility of non-professional modalities such as the 12-step fellowships and mutual-help groups in the communities. Understanding the role and impact of these peer support networks could offer valuable insights into tailoring effective interventions that address the unique needs of individuals in the gay men community on their journey towards recovery.

## Conclusions

The present study is the first to explore trajectories and factors associated with drug use and the challenges individuals face in sustaining abstinence from drug use among a sample of substance use treatment-experienced clients in Singapore. It revealed several themes that is of clinical interest, specific to drug use behaviors, motivations toward drug cessation, and personal narratives surrounding one’s recovery journey. To this end, the findings could be used to review and bolster the effectiveness of existing interventional programs and organizational policies in facilitating drug use recovery in the community.

### Supplementary Information

**Supplementary Material 1.** 

## Data Availability

The datasets generated and/or analysed during the current study are not publicly available due to potential legal risks to participants and clients to The Greenhouse Community Services Limited but are available from the corresponding author on reasonable request.

## Abbreviations

CCS: Case Closing Summary
CPR: Care Plan Review
PN: Progress Notes
RA: Research Assistant
