# Supplementary material for: Navigating drug use, cessation, and recovery: a retrospective case notes review among sexual minority men at a community-based service in Singapore
Source: Subst Abuse Treat Prev Policy. 2024 Apr 16;19:23. doi: 10.1186/s13011-024-00605-x (PMC11020317; doi:10.1186/s13011-024-00605-x)
Supplement: Supplementary file 1 — Supplementary Material 1. [file 13011_2024_605_MOESM1_ESM.docx]

**Supplementary Material**

**Supplementary Table 1**

*Theme Descriptions for Maintaining Stability, Establishing Healthy Relationships, and Recovery Struggles and Barriers for Table 3*

| **Category** | **Theme** | **Theme Description** |
| --- | --- | --- |
| Maintaining stability | Self-security | When clients express the want to have an open and non-judgmental acceptance of their own weakness^a^ |
|  | Self-discovery | When clients mention that they want to gain a deeper understanding of who they are, their values, needs and emotions |
|  | Self-awareness | When clients express that they want to better identify their state and emotions in the present moment. |
|  | Self-doubt | When clients have an unstable self-view, and expresses uncertainty in their own abilities |
|  | Self-esteem | When clients assess people and what they have accomplished to their values and goals and express their wants to boost their confidence and self-esteem^a^ |
|  | Self-development | When clients share about some things that they hope to work on, in order to better themselves |
|  | Self-efficacy | When clients express their want to be self-sufficient or self-sustaining to reach specific goals. |
|  | Reevaluating values | When clients reflect on personal values and reassess expectations of themselves in living according to their values |
|  | Trait anxiety | A stable personality characteristic, when cts have a tendency to respond with concerns, worries or troubles to various situations. Trait anxiety can also be associated with psychological conditions that reflect a constant high arousal^b^ |
|  | Desire for an ideal self | When the client talks about desires to achieve an ideal version of themselves, which may or may not be realistic |
|  | Suicidal ideation | When the clients express thoughts about suicide |
|  | Cognitive reframing | When clients attempt to reframe their mindset to make sense of their reality^c^ |
|  | Managing emotions & feelings | When clients mentions that they want to improve emotional regulation, but does not specify the emotion/feeling |
| Establishing healthy relationships | Relationship codependency | When clients mention that both parties in the relationship are over-reliant on each other to satisfy each other's needs (e.g feeling accepted). These kinds of relationship may involve partners enabling one another to engage in risky behaviour (e.g using or risky sex) |
|  | Establishing social support | When clients distinguish the kinds of social support they want to create with others (e.g friends, family, peers), or ways to improve the quality of current support |
|  | Setting healthy boundaries | When clients want to create a space where they can love themselves and others simultaneously. This includes taking another person’s feelings into consideration, setting clear limits and showing mutual respect^a^ |
|  | Managing relationship tensions | When clients are struggling with tensions or conflict in relation to (i) either their birth or affinal family relationship; (ii) Intimate partner relationship; or (iii) friendships |
|  | Grieving loss of intimate relationship | When clients mention that they are grieving a relationship loss that happened in the past/recently |
|  | Social pressure to recovery | When clients share about the social pressures, they face to seek recovery services |
|  | Desires for authentic connection | When clients distinguish the the kinds of social connection they want to create with others (e.g friends, family, peers), or ways to improve the quality of current relationships. |
| Recovery struggles and barriers | Fears and worries | When clients share about fears and worries that they have regarding individual counselling, or in their lives |
|  | Trauma and triggers | When clients mentions that they want to process feelings or past traumatic situations/experiences in a counselling setting. This also includes potential triggers of trauma. |
|  | Addressing symptoms of anxiety/panic | When clients practice specific techniques during counselling to help them cope with symptoms of anxiety |
|  | Attempts to overcome Struggles | When clients work with the counsellor to address other recovery struggles, such as role-playing or experimentation |
|  | Religious issues | When clients re-evaluate their religious identity and values, attempting to make peace with the conflicting narratives they are faced with |
|  | Preparing for post-therapy | When clients share about their plans and preparations for post-counselling |

^a^Brown, B. (2021). Atlas of the heart: Mapping meaningful connection and the language of human experience. Random House.

^b^Gidron, Y. (2013). Trait anxiety. Encyclopedia of behavioral medicine, 1, 1989.

^c^Robson Jr, J. P., & Troutman-Jordan, M. (2014). A concept analysis of cognitive reframing. Journal of Theory Construction & Testing, 18(2).

^d^Braslow, M. D., Guerrettaz, J., Arkin, R. M., & Oleson, K. C. (2012). Self‐doubt. Social and Personality Psychology Compass, 6(6), 470-482.

**Supplementary Table 2**

*Full Progress Notes for The Case Study*

| **Session No.** | **Goals and Motivations to stop drugs** | **Relapse** | **Maintaining Stability** | **Establishing Healthy Relationships** | **Recovery Struggles/Barriers** |
| --- | --- | --- | --- | --- | --- |
| 1 | “Trying to be clean” (CC) | 22.08.21.  Feeling “liberated” after substance-use relapse | **Lack of self-security:**   - Concerns with “deficient self” and struggles with multiple addictions. | **Relationship codependency:**   - Difficulties in managing co-dependent relationship.   **Establishing social support:**   - Need for stabilisation and peer support, to address very recent relapse. | **Fears and worries:**   - Fearful about listening more compassionately to self. |
| 2 |  | - | **Managing emotions and feelings:**   - Safety planning for suicidality | **Setting healthy boundaries:**   - Boundary work in relationships - Clarified locus of control in relationships   **Establishing social support:**   - Planned social support for suicidality/relapse. - Need to review relapse management and relationship with peer supporter. | **Trauma and triggers:**   - Examined childhood trauma, and self-image as “monster” with dark side - Psychoeducation on trauma’s impact and therapy process |
| 3 | Managing cravings:  Mild panic attack mid-session when craving arose |  | **Managing emotions and feelings:**   - Acknowledged emotional avoidance, fear of loneliness.   **Self-security:**   - Desire to “get rid of” old and bad self - Psychoeducation on self-acceptance and motivation. Challenging good/bad new/old dichotomy | **Establishing social support:**   - Identified new strength in finding social support. - Interest in peer support groups. | **Fears and worries:**   - Fear of ‘losing edge’ - Feeling exposed to others   **Addressing symptoms of anxiety/panic:**   - Grounding to address panic. Reviewing coping strategies and relapse prevention |
| 4 |  | 14.09.21  leading to sleeplessness and increased porn use | **Managing emotions and feelings:**   - Feeling “lost” in transition - Identifying dilemmas underlying “lost” feelings.   **Trait anxiety:**   - Anxiety over relationship with partner   **Self-security:**   - Increased self-blame | **Relationship codependency:**   - Identified co-dependent patterns   **Setting healthy boundaries:**   - Clarifying relationship boundaries and needs.   **Establishing social support:**   - Planned new resources in social support and stabilisation. | **Fear and worries:**   - Experienced fears of losing drive, scapegoating father, being selfish |
| 5 |  |  | **Self-discovery:**   - Clarified what is valuable about “old self”   **Managing emotions and feelings:**   - Explored needs underlying anger   **Self-security:**   - Amplified ambivalence over self-acceptance | **Managing relationship tensions:**   - Upset over separation from partner; poor sleep with rumination - Identified longing for companionship and desire to control partner. - Defined meaning of this “bonus time” of separation. |  |
| 6 | **Cravings:**   - Desire to use 3 months well. |  | **Managing emotions and feelings:**   - Anger over self- acceptance   **Managing emotions and feelings:**   - Noting meanings of anger   **Cognitive reframing:**   - Nurturing spiritual hope and recognising God’s “joke” | **Managing relationship tensions:**   - Clarified ambivalence over partner’s new independence | **Attempts to overcome struggles:**   - Explored meanings of being stalled. - Defined values to guide next 3 months.   **Fears and worries:**   - Identified fear of being “soft” and “yielding”, and desire to honour father’s disciplining |
| 7 |  |  | **Self-security:**   - Distinguished fallible self vs. deficient self. | **Setting healthy boundaries:**   - Examined relationship boundaries and expectations in family   **Managing relationship tensions:**   - Struggling over loss of moral authority in family. | **Fears and worries:**   - Losing motivation to change - Conflicted relationships with family and church friends   **Religious issues:**   - Clarified values in religious identity   **Attempts to overcome struggles:**   - Designed experiment in “showing up” when avoidant - Explored ambivalence about change |
| 8 |  |  | **Managing emotions and feelings:**   - Feeling numb - Distracting self with porn and casual sex. - Experiencing distress underlying numbness.   **Self-security:**   - Addressed harsh self-criticism over “bad self”. - Recognised some common humanity in good/bad selves   **Suicidal ideation:**   - 17.10.2021. Sister and mother contacted; client declined to check into IMH or seek support from medical social worker.   **Self-discovery:**   - Aware of “joyless” distractions | **Grieving loss of intimate relationship:**   - Grief over separation from partner.   **Establishing social support:**   - Planned support in case of suicidal ideation | **Fear and worries:**   - Identified fear of losing partner and struggle over letting go |
| 9 | **Managing recovery expectations:**   - Exploring desire for “quick fix” to addictions. - Review of progress; psychoeducation on therapeutic alliance. - Noting choices amid compulsion. |  | **Managing emotions and feelings:**   - Feelings of despair - Managing compulsive behaviours   **Self-awareness:**   - Recognised less avoidance of sorrow and more “slowing down” | **Establishing social support:**   - Acknowledged value of collaboration.   **Setting healthy boundaries:**   - Identified decision points and intimacy needs during compulsive episodes. | **Fears and worries:**   - Concerns about therapeutic progress - Processed fear of suffering. |
| 10 |  |  | **Managing emotions and feelings:**   - Avoidance behaviours   - Distinguished avoidance/distraction behaviours. - Feeling “lost” at home   **Self-discovery:**   - Developed explanations of therapeutic needs and progress - Identified examples of psychological flexibility | **Social pressure to recovery:**   - Pressure from others to be “cured”   **Managing relationship tensions:**   - Setting relationship goals in family - Prioritised relationship with children | **Attempts to overcome struggles:**   - Role-play on responses to pressures |
| 11 |  |  | **Managing emotions and feelings:**   - “Auto-pilot” behaviours. - Unclarity on values. - Feeling joy in play and humour.   **Self-awareness:**   - Developing curiosity about behaviours.   **Building self-esteem:**   - Experiencing joy in values | **Establishing social support:**   - Planned to join recovery community. | **Fears and worries:**   - Uncertainty over end of therapy - Psychoeducation on phases of change and termination. |
| 12 |  |  | **Managing emotions and feelings:**   - Anxiety about facing world with vulnerable self - Acknowledged value of “balance”, especially in distraction/defusion.   **Self-discovery:**   - Exploring voices of moral guidance - Experimenting with values - Developing perspective on self | **Managing relationship tensions:**   - Managing family dynamics   **Desires for authentic connection:**   - Identified desire to come out to older children. - Appreciated courage and desire to be more authentic. |  |
| 13 | **Managing triggers:**   - Managing triggers in recovery - Clarifying meaning of triggers. |  | **Building self-esteem:**   - Re-valued humour, honesty, freedom.   **Managing emotions and feelings:**   - Heightened anxiety about triggers. | **Desires for authentic connection:**   - Coming out to children - Examining values in coming out to family   **Managing relationship tensions:**   - Increasing conflict with partner. - Exploring hurt feelings in marriage; - psychoeducation on couples therapy - Clarified “unspeakable injustice” and anxieties in marriage.   **Setting healthy boundaries:**   - Developed idea of “training zone”; balance between self-reliance and social support. |  |
| 14 | **Re-evaluating recovery expectations:**   - Psychoeducation on sleep hygiene and recovery stages. |  | **Re-evaluating values:**   - Acknowledged need for patience. - Identified values-based actions.   **Self-doubt:**   - “Seeds of doubt” about recovery. - Exploring self-doubt and choice points.   **Managing emotions and feelings:**   - Practised “Dropping Anchor” | **Managing relationship tensions:**   - Struggling over “double standards” in marriage - Amplified ambivalence over boyfriend’s resourcefulness - Managing family tensions.   **Setting healthy boundaries:**   - Frustrated with partner’s boundaries. - Examining stances in relationships. |  |
| 15 |  |  | **Managing emotions and feelings:**   - Calm demeanour - Anxiety over resuming relationship with partner - Listening to “need to rebel” - Practised self-kindness - Increased self-compassion for “healing brain”.   **Self-discovery:**   - Clarifying sources of anxiety | **Establishing social support:**   - Distinguished social support/rigid rules in recovery.   **Setting healthy boundaries:**   - Identified desire for control in relationship; - rehearsed being more vulnerable to partner. | **Attempts to overcome struggles:**   - Resistance in recovery - Balancing work and family |
| 16 |  |  | **Managing emotions and feelings:**   - Tired demeanour after some restless sleep. - Anxiety over new experiences, including job interviews and recovery meetings - Guilt over marriage - Reviewing “Dropping Anchor” - Used breath/body in cognitive expansion.   **Re-evaluating values:**   - Clarifying value of authenticity to self. - Re-valued “showing up” as vulnerable self for interviews and recovery meetings.   **Self-development:**   - Practised self-compassion exercise for relationships | **Desires for authentic connection:**   - Desire for more authenticity.   **Setting healthy boundaries:**   - Planned compassionate letters to partner. |  |
| 17 |  |  | **Managing emotions and feelings:**   - Lucid reflections. Calm demeanour. - Acknowledged guilt from “objectively disordered” label - Exploring sources of termination anxiety.   **Self-doubt:**   - Practising “Yes, and…” for self-doubt and sexual desire.   **Re-evaluating values:**   - Connected self-doubt with value of authenticity. - Challenging assumptions about gay promiscuity. | **Desires for authentic connection:**   - Need for “clean” “untainted” relationships with other gay men. - Desire for gay friendship.   **Establishing social support:**   - identified new sources of peer support.   **Setting healthy boundaries:**   - Mutual gratitude for therapeutic alliance |  |
| 18 | **Triggers and cravings:**   - Rising “temptations” to relapse. |  | **Managing emotions and feelings:**   - Anxious demeanour after “week of turmoil” - Practising compassion for “imperfect self”. - Clarifying sources of anxiety.   **Desires for an ideal self:**   - Desire to be “absolutely good”   **Self-discovery:**   - Expanded room for multiplicity in self | **Setting healthy boundaries:**   - Testing therapeutic boundaries - Softened expectations of partner after 3-month break | **Fears and worries:**   - Experiencing fear of termination |
| 19 |  |  | **Managing emotions and feelings:**   - Calm and lucid reflections. - Managing anti-depressants. - Juggling “multiple selves”. - Reviewing strategies developed by different parts in self. - Increased perspective on “healing brain” in recovery. - Gratitude for creativity and resilience in self. | **Setting healthy boundaries:**   - Frustration over partner’s boundaries. - Exploring value in relationship boundaries. - Appreciation for partner’s needs and growth; insight into their “perpetual conflict”. | **Preparing for post-therapy:**   - Co-designing termination activity. |
| 20 | **Re-evaluating recovery plans:**   - Reviewing therapeutic journey |  | **Managing emotions and feelings:**   - Excited demeanour. Lucid reflections. - Self-blame over relationship boundaries - Anxiety about being a new leader   **Self-sufficiency:**   - Self-compassion practice for self and other - Balanced self-doubt with self-kindness and humour. - Honoured integration of good/bad new/old parts. | **Establishing social support:**   - Need for “more therapy”   **Desires for authentic connection:**   - Experimenting with authenticity in leadership - Discerned growth of “imperfect self” in new community   **Setting healthy boundaries:**   - Receiving mutual gratitude. |  |

*Note:* Columns represent overall categories, recovery themes are bolded in the table, and raw data from clinical notes are in bullet points, arranged according to the themes they represent.
